# Autoimmune Pancreatitis Masquerading as Pancreatic Cancer: A Case Report and Literature Review

**DOI:** 10.7759/cureus.21900

**Published:** 2022-02-04

**Authors:** Khushboo K Agarwal, Ripsy Jassal, Alexander Browne, Mohammed Hossain, Reza Akhtar

**Affiliations:** 1 Internal Medicine Department, Jersey Shore University Medical Center, Neptune, USA; 2 Family Medicine, University of Arkansas for Medical Sciences North Central Family Medicine Residency, Batesville, USA

**Keywords:** autoimmune pancreatitis (aip), steroid use, pancreatic cancer

## Abstract

Autoimmune pancreatitis (AIP) is a chronic fibro-inflammatory disease of the pancreas that belongs to the spectrum of immunoglobulin G-subclass 4-related diseases (IgG4-RD). It is sometimes associated with a visible pancreatic mass mimicking pancreatic cancer on imaging. The most common presentations include abdominal pain and obstructive jaundice in elderly men. Similar to other IgG4-RD, it can cause cholangiopathy, nephritis, orbital pseudotumor, and extensive lymphadenopathy. Here, we present the case of a 53-year-old female with abdominal pain and obstructive jaundice, which was diagnosed as AIP in association with significantly elevated tumor marker carbohydrate antigen 19-9 (CA 19-9). She responded to biliary decompression and steroid treatment, potentially avoiding extensive surgical intervention. On follow-up, her CA 19-9 and IgG4 levels were normalized. AIP should be high on the differential diagnosis during the evaluation of a pancreatic mass.

## Introduction

Autoimmune pancreatitis (AIP) is a rare form of recurrent acute pancreatitis [1]. As a part of systemic immunoglobulin G-subclass 4 (IgG4)-related sclerosing diseases, it is characterized by chronic lymphoplasmacytic inflammation of the pancreas with irregular narrowing of the main pancreatic duct and hypergammaglobulinemia [2,3]. It is sometimes associated with a visible pancreatic mass mimicking pancreatic cancer on imaging. Differentiating these entities is crucial owing to the obvious differences in treatment course and prognosis. Here, we present a case of acute AIP presenting with obstructive jaundice and pancreatic mass on imaging studies responsive to steroids [4].

## Case presentation

A 53-year-old female presented to the emergency department with the chief complaints of worsening epigastric pain and intermittent vomiting for three weeks. The pain was severe, intermittent, radiating to the back, and worsened post-prandially. The patient also endorsed unintentional weight loss and dark urine. She denied dyspnea, chest pain, fevers, or headaches. Social history was pertinent for 30-pack-year smoking history and occasional alcohol use of three drinks per week. She denied any history of pancreatic or hepatobiliary disease or cancer in herself or her family.

Her physical examination was pertinent for jaundice and scleral icterus. Epigastric tenderness without hepatosplenomegaly was noted on abdominal examination. Her laboratory data showed hemoglobin of 12.6 g/dL, creatinine 0.6 mg/dL, aspartate transaminase 323 IU/L, alanine transaminase, 409 IU/L, total bilirubin 6 mg/dL, alkaline phosphatase 333 U/L, lipase 68 U/L, amylase 32 U/L, and carbohydrate antigen 19-9 (CA 19-9) of 2,592 U/mL. Her international normalized ratio was 2.09 (Table 1).

**Table 1:** Summary of laboratory investigations on admission, day one follow-up before EUS/ERCP, day three follow-up after CBD stent, day 21 follow-up after steroid treatment, and day 40 follow-up after steroid treatment. CA 19-9: carbohydrate antigen 19-9; ALK: alkaline phosphatase; AST: aspartate aminotransferase; ALT: alanine transaminase; ANA: antinuclear antibody; IgG4: immunoglobulin G-subclass 4; IgG: immunoglobulin G; ND: not done; CBD: common bile duct; EUS: endoscopic ultrasound; ERCP: endoscopic retrograde cholangiopancreatography

	On admission	Day 1 follow-up before EUS/ERCP	Day 3 follow-up after CBD stent	Day 21 follow-up after steroid treatment	Day 40 follow-up after steroid treatment	Reference values
CA 19-9	2,592	3,295	938	48.9	NA	0–35 U/mL
Total bilirubin	9.1	11.3	3.7	1.3	0.8	0.2–1.3 mg/dL
ALK	271	292	355	74	57	38–126 U/L
AST	325	323	194	40	27	10–42 U/L
ALT	408	452	324	57	33	10–60 U/L
ANA	ND	ND	1.08	ND	ND	≤0.90 AU
IgG	ND	ND	2,010	ND	ND	Adults: 600–1,560 mg/dL
IgG4	ND	ND	71	ND	ND	1-123 mg/dL

**Figure 1:**
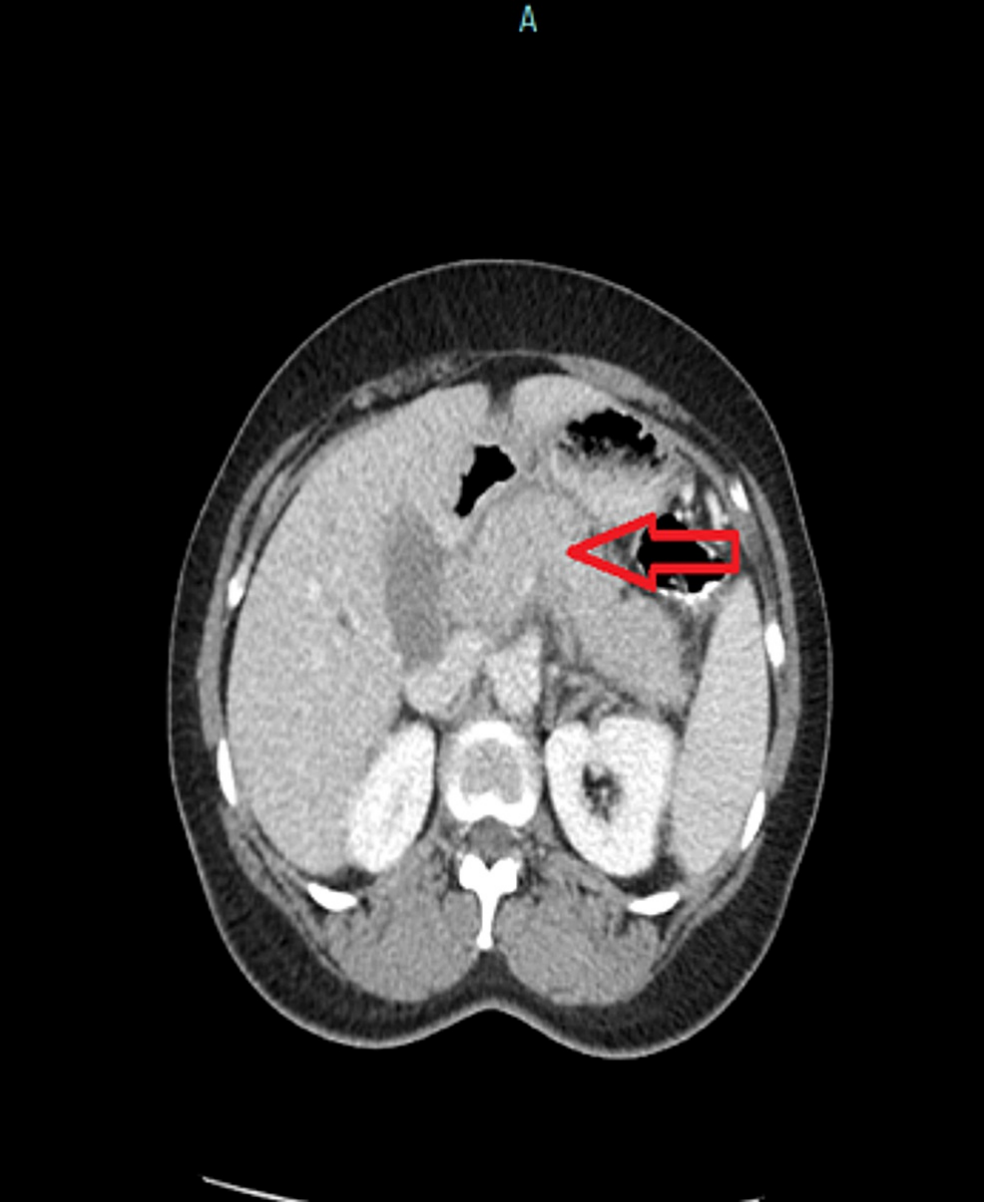
Pre-steroid treatment CT of the abdomen: pancreatic head and uncinate process appear enlarged, although no discrete enhancing pancreatic mass was identified (red arrow). CT: computed tomography

**Figure 2:**
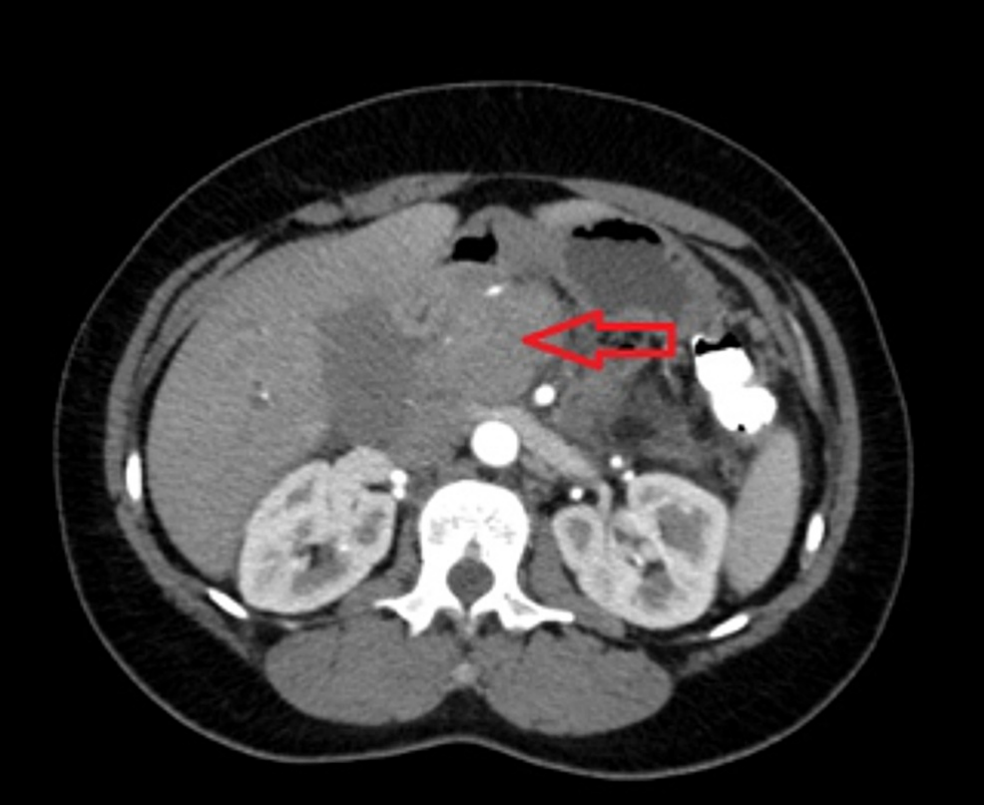
Pre-steroid treatment CT angiogram of the chest and abdomen revealing enlarged pancreatic head/uncinate process (red arrow) along with soft tissue density extending inferiorly and measuring 4.0 × 3.7 cm. CT: computed tomography

An abdominal ultrasound showed a 3 cm pancreatic mass with marked intrahepatic duct and common bile duct (CBD) dilatation up to 15 mm distally. Computed tomography (CT) of the abdomen and pelvis showed an enlarged pancreatic head and uncinate process without a discrete enhancing mass. No pancreatic duct dilation was noted. Intrahepatic duct dilation and CBD of 13 mm in diameter were noted (Figure 1). CT angiogram of the abdomen and pelvis showed that the pancreatic head/uncinate process was enlarged along with soft tissue density extending inferiorly, which enhanced similar to the remainder of the tissue measuring 4.0 × 3.7 cm. On coronal imaging, it measured approximately 5.1 cm in the cephalocaudal dimension (Figures 1, 2).

**Figure 3:**
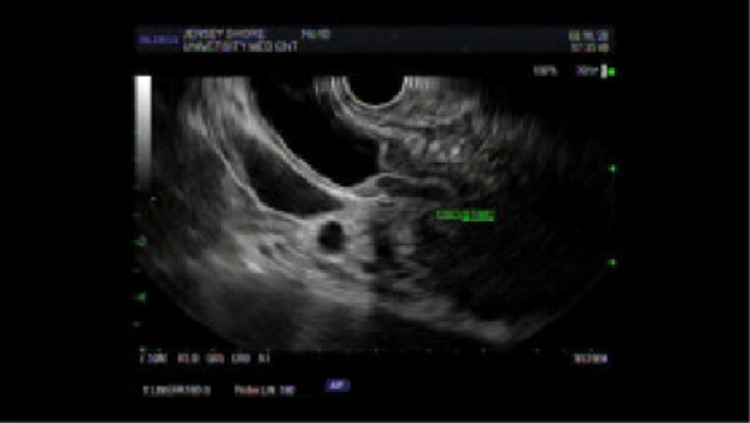
Endoscopic ultrasound of the common bile duct stricture.

MRI abdomen revealed a sausage-shaped appearance of the pancreas with intra and extrahepatic ductal dilatation. Endoscopic ultrasound (EUS) revealed an ill-defined pancreatic head mass compressing the CBD (Figure 3). EUS fine-needle aspiration was performed and was negative for malignancy. Endoscopic retrograde cholangiopancreatography (ERCP) revealed a long CBD stricture causing intrahepatic dilation. Cytology brushings of the CBD/pancreas/duodenum were obtained and were negative for malignant cells. Duodenal mucosa showed marked acute and chronic inflammation. Clinically, the duodenum/ampulla biopsy revealed no malignancy. A plastic stent was placed in the CBD for decompression. Peri-ampullary biopsies of the duodenum were notable for positive IgG4 immunostain at approximately 28/high-power field (HPF) (reference range: >50/HPF and or IgG4/IgG cells >40%).

**Figure 4:**
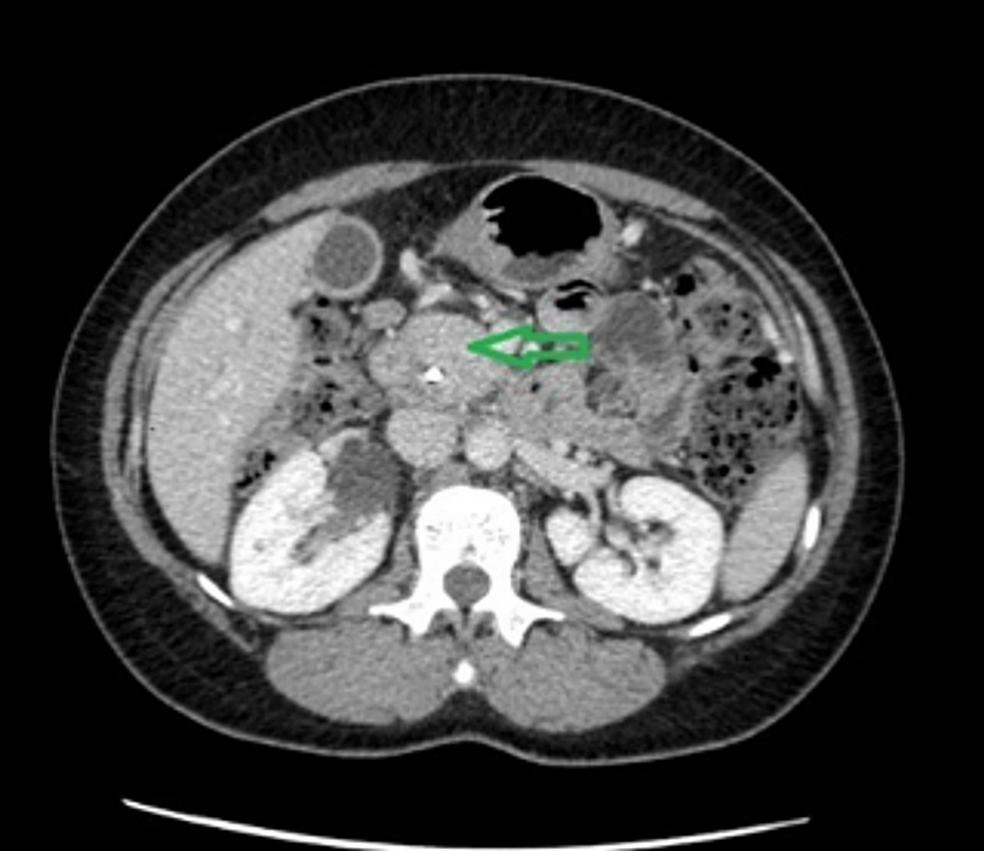
On day 21 follow-up post-steroid treatment, CT angiogram of the chest and abdomen revealed a pancreatic head measuring approximately 3.2 × 1.6 cm, an improvement from 4.3 × 3 cm. The pancreatic body (green arrow) at the current examination measured approximately 1.8 cm in its thickness improving from 2.8 cm. CT: computed tomography

AIP was suspected based on imaging characteristics of a sausage-shaped pancreas (Figure 2), negative fine-needle aspiration, and positive IgG4 staining on peri-ampullary biopsy. After prednisone therapy, clinical and radiologic improvement along with a significant decrease in her tumor markers elevated AIP in her differential diagnosis. The patient was started on a tapering course of prednisone. Follow-up imaging after 21 days revealed improvement of the pancreatic head enlargement from 4.3 × 3 cm to 3.2 × 1.6 cm (Figures 2, 4), as well as significant improvement in laboratory values (Table 1). Clinically, the patient remained symptom-free and tolerated her course of prednisone without any side effects.

## Discussion

AIP is a chronic fibro-inflammatory disease of the pancreas that belongs to the spectrum of immunoglobulin G-subclass 4-related diseases (IgG4-RD) [5]. The term AIP was first introduced by Yoshida et al. in 1995 [6]. It is common among elderly men, with a mean age of diagnosis above 60 years. It is more predominant in males, with a male-to-female ratio of 3:1 [7]. The incidence of AIP is 1:100,000 in the general population [7]. Clinically, AIP presents with obstructive jaundice (75%) and abdominal pain (68%) [8,9]. Similar to other IgG4-RD, other organs may be involved, leading to cholangiopathy, nephritis, orbital pseudotumor, and extensive lymphadenopathy. Their presence may serve as additional diagnostic clues [8]. Our patient had a classic presentation of subacute abdominal pain and obstructive jaundice without the involvement of other organs. IgG4 is a serologic marker for AIP and can be elevated in 10% of pancreatic cancer patients [10,11]. Elevated levels of IgG4 support the diagnosis of AIP. However, normal levels do not rule out the possibility of AIP.

AIP is subdivided into two types: type 1 and type 2. In type 1 AIP, also known as lymphoplasmacytic sclerosing pancreatitis, the pancreas is affected as part of systemic IgG4-positive disease. Type 2 AIP is characterized by histologically confirmed idiopathic duct-centric pancreatitis, often presenting with granulocytic epithelial lesions with or without granulocytic acinar inflammation along with absent IgG4-positive cells (0-10 cells per HPF), as well as without systemic involvement [10]. Neither were revealed in our patient’s biopsy.

There are multiple guidelines to diagnose AIP, including those recommended by the Japanese Pancreas Society (JPS; JPS-2006 and JPS-2011), Korean Criteria, Asian Criteria, HISORt (histology, imaging, serology, response to corticosteroids), and, most recently, the International Consensus Diagnostic Criteria (ICDC). The JPS guidelines require ERCP as part of its diagnostic criteria [10]. Our patient underwent an ERCP as part of her diagnostic workup with consistent findings.

Pancreatic imaging abnormalities are found in up to 85% of patients with AIP [12]. The diagnostic features of AIP on CT or MRI include diffuse parenchymal enlargement with delayed enhancement, sometimes associated with rim-like enhancement. Indeterminate imaging of the pancreas includes segmental or focal enlargement with delayed enhancement [13,14]. In our patient, the CT scan showed an enlarged pancreatic head/uncinate process without a discrete mass. MRI of the abdomen showed a sausage-shaped appearance of the pancreas with intra and extrahepatic ductal dilatation (Figure 4). EUS findings typically include pancreatic parenchymal abnormalities in the entire pancreas comprising diffuse hypoechogenicity and hyperechoic strands. The overall size of the pancreas appears to be enlarged, measuring approximately 41.6 mm × 30.4 mm in the maximal cross-sectional diameter (Figure 3).

ERCP and magnetic resonance cholangiopancreatography ductal imaging features can provide additional evidence in diagnosing AIP. According to the ICDC guidelines, typical features on ERCP are long (>one-third the length of the main pancreatic duct) or multiple strictures of the pancreatic duct without marked upstream dilatation [15]. Other nonspecific features include segmental/focal narrowing of the main pancreatic duct without marked upstream dilatation (duct size <5 mm). Additionally, a focal stricture of the proximal or distal CBD or irregular narrowing of the intrahepatic ducts can be noted [16]. Our patient’s ERCP showed long CBD stricture causing intrahepatic dilation.

Diagnosis is supported by serological markers (Table 2). A two-fold increase in serum IgG4 level is strongly associated with AIP [15,16].

**Table 2:** IgG4 levels and the associated sensitivity and specificity. IgG4: immunoglobulin G-subclass 4

IgG4 level	Sensitivity	Specificity
>280 mg/dL	53%	99%
>140 mg/dL	76%	93%
>1,800 mg/dL	43%	88%

Notably, IgG4 can be elevated in 10% of pancreatic cancer patients [16]. Specificity improves to 100% when serum IgG4 level increased to 270 mg/dL (twice the upper limit of normal) [16]. Our patient had IgG and IgG4 levels of 2,010 mg/dL and 71 mg/dL, respectively.

Serologic markers are used to support the diagnosis. Approximately 25% of AIP patients show increased levels of CA 19-9 at >37 U/mL, and approximately 12.2% of patients show increased levels of >100 U/mL [11,17]. Our patient had a higher CA 19-9 initially which decreased significantly after biliary decompression (Table 1).

Early corticosteroid therapy is indicated in patients with AIP unless otherwise contraindicated [17]. Patients who receive prednisone 0.6 mg/kg achieved a remission rate of 98%. The relapse rate is 23% in patients on steroids and 32% in patients who do not receive steroids [18]. There is no difference in outcomes between high or low-dose steroids [18,19]. Our patient was on a prednisone taper for over three months.

Steroid therapy should only be initiated after pancreatic malignancy has been ruled out, including a negative EUS-guided fine needle aspiration. For a diagnostic corticosteroid trial to be considered positive, a rapid (within two weeks) improvement in radiologic pancreatic/extrapancreatic manifestations should be noted [15], as seen in our patient. Spontaneous resolution of symptoms or radiologic features in patients with AIP without the use of steroid treatment, which is occasionally seen, is not considered a diagnostic criterion according to the ICDC guidelines. Overall, improvement in symptoms or decreased CA 19-9 levels should not be the only evidence for the diagnosis of AIP in the absence of other diagnostic features. CA 19-9 levels in our patient decreased.

## Conclusions

Elevated CA 19-9 levels often lead to a diagnosis of pancreatic cancer; however, as seen in this patient, other differentials must also be considered. The patient had a significant elevation of her CA 19-9 initially, which was likely due to CBD dilation. Further clinical workup with serology, imaging, and histopathology revealed AIP. We, subsequently, treated her with biliary decompression and steroids, which proved effective, and the patient significantly improved. Further workup and treatment for AIP avoided both a pancreatic cancer diagnosis and unnecessary surgical intervention.
